# The Mobile Solutions for Immunization (M-SIMU) Trial: A Protocol for a Cluster Randomized Controlled Trial That Assesses the Impact of Mobile Phone Delivered Reminders and Travel Subsidies to Improve Childhood Immunization Coverage Rates and Timeliness in Western Kenya

**DOI:** 10.2196/resprot.5030

**Published:** 2016-05-17

**Authors:** Dustin G Gibson, E. Wangeci Kagucia, Benard Ochieng, Nisha Hariharan, David Obor, Lawrence H Moulton, Peter J Winch, Orin S Levine, Frank Odhiambo, Katherine L O'Brien, Daniel R Feikin

**Affiliations:** ^1^ Department of International Health Johns Hopkins Bloomberg School of Public Health Baltimore, MD United States; ^2^ Health and Demographic Surveillance System Branch Kenya Medical Research Institute and Centers for Disease Control and Prevention Public Health Collaboration Kisian Kenya; ^3^ Division of Viral Diseases Centers For Disease Control and Prevention Atlanta, GA United States

**Keywords:** text message, reminders, SMS, M-PESA, Kenya, mobile, conditional cash transfer, CCT, incentive, measles, mHealth, immunization, pentavalent

## Abstract

**Background:**

Text message (short message service, SMS) reminders and incentives are two demand-side interventions that have been shown to improve health care–seeking behaviors by targeting participant characteristics such as forgetfulness, lack of knowledge, and transport costs. Applying these interventions to routine pediatric immunizations may improve vaccination coverage and timeliness.

**Objective:**

The Mobile Solutions for Immunization (M-SIMU) trial aims to determine if text message reminders, either with or without mobile phone–based incentives, sent to infant’s parents can improve immunization coverage and timeliness of routine pediatric vaccines in rural western Kenya.

**Methods:**

This is a four-arm, cluster, randomized controlled trial. Villages are randomized to one of four study arms prior to enrollment of participants. The study arms are: (1) no intervention (a general health-related text message will be texted to this group at the time of enrollment), (2) text message reminders only, (3) text message reminders and a 75 Kenyan Shilling (KES) incentive, or (4) text message reminders and a KES200 incentive. Participants assigned to study arms 2-4 will receive two text message reminders; sent 3 days before and one day before the scheduled immunization visit at 6, 10, and 14 weeks for polio and pentavalent (containing diphtheria, tetanus, pertussis, hepatitis B, and *Haemophilus influenza* type b antigens) type b antigens) vaccines, and at 9 months for measles vaccine. Participants in incentive arms will, in addition to text message reminders as above, receive mobile phone–based incentives after each timely vaccination, where timely is defined as vaccination within 2 weeks of the scheduled date for each of the four routine expanded program immunization (EPI) vaccination visits. Mother-infant pairs will be followed to 12 months of age where the primary outcome, a fully immunized child, will be ascertained. A fully immunized child is defined as a child receiving vaccines for bacille Calmette-Guerin, three doses of pentavalent and polio, and measles by 12 months of age. General estimating equation (GEE) models that account for clustering will be employed for primary outcome analyses.

**Results:**

Enrollment was completed in October 2014. Twelve month follow-up visits to ascertain immunization status from the maternal and child health booklet were completed in February 2016.

**Conclusions:**

This is one of the first studies to examine the effect of text message reminders on immunization coverage and timeliness in a lower income country and is the first study to assess the effect of mobile money-based incentives to improve immunization coverage.

**Trial Registration:**

Clinicaltrials.gov NCT01878435; https://clinicaltrials.gov/ct2/show/NCT01878435 (Archived by WebCite at http://www.webcitation.org/6hQlwGYJR)

## Introduction

The decade from 2010 to 2019 has been dubbed the “Decade of Vaccines” with renewed focus on immunization by major international groups like World Health Organization, United Nations Children's Emergency Fund, the Global Alliance for Vaccines, and the Bill and Melinda Gates Foundation [1-3]. A key component in the Global Vaccine Action Plan is the recognition that both demand- and supply-side deficiencies need to be addressed in order to achieve universal immunization [4]. Ensuring more infants receive timely vaccination is a major component in efforts to reduce child mortality by two-thirds and achieve Millennium Development Goal 4 [5]. Every year, immunization programs are estimated to save over 2.5 million lives globally [6], with the majority of deaths averted occurring in Africa [7].

Timely vaccination is important for several reasons. First, the diseases that pediatric vaccines protect against often have highest morbidities and mortalities at earlier stages of life. Delays of infant immunization have been associated with increased cases of pertussis [8,9], hepatitis B [10], and *Haemophilus influenza* type b [11]. Second, timely vaccination heightens population herd immunity levels [12], thereby protecting those that are too young to be vaccinated or are medically contraindicated. Delays in vaccination lessen population coverage and create a pool of susceptible individuals, thereby increasing the pathogen’s ability to spread and theoretically increasing the risk of exposure.

Interventions to improve immunization coverage and timeliness are important because approximately 1 in 13 children in Kenya and 1 in 5 children in our study site, Siaya County in western Kenya, will die before their 5th birthday, with the majority of deaths attributed to infectious diseases [13,14]. Many of these deaths are preventable by vaccination, yet many children in Kenya are not vaccinated or are vaccinated late [15,16].

Two demand-side interventions, text message (short message service, SMS) reminders [17-22] and small monetary incentives [23-25], have been shown to motivate positive health behaviors in resource constrained settings. Moreover, text message reminders have been shown to modestly improve immunization coverage in the United States [26-29]; however, the efficacy of text message reminders to improve immunization coverage in sub-Saharan Africa, until recently [30,31], has neither been evaluated nor used in conjunction with monetary incentives. This randomized controlled trial will test whether text messaging reminders, either with or without mobile phone-based incentives, can improve timeliness and coverage of routine pediatric immunizations.

Critically, the success of our pilot study in a neighboring division, Karemo, shows that a mobile phone-based system that delivers incentives and text messaging reminders is technically feasible and welcomed by the community [32]. Lessons and challenges learned from the pilot study will be incorporated into the design of the current cluster randomized controlled trial.

The Mobile Solutions for Immunizations (M-SIMU) study is a four-arm, cluster, randomized controlled trial, which evaluates the impact of providing caregivers text message reminders and monetary incentives on the proportion of children that are fully vaccinated by 12 months of age.

## Methods

### Study Design

**Figure 1:**
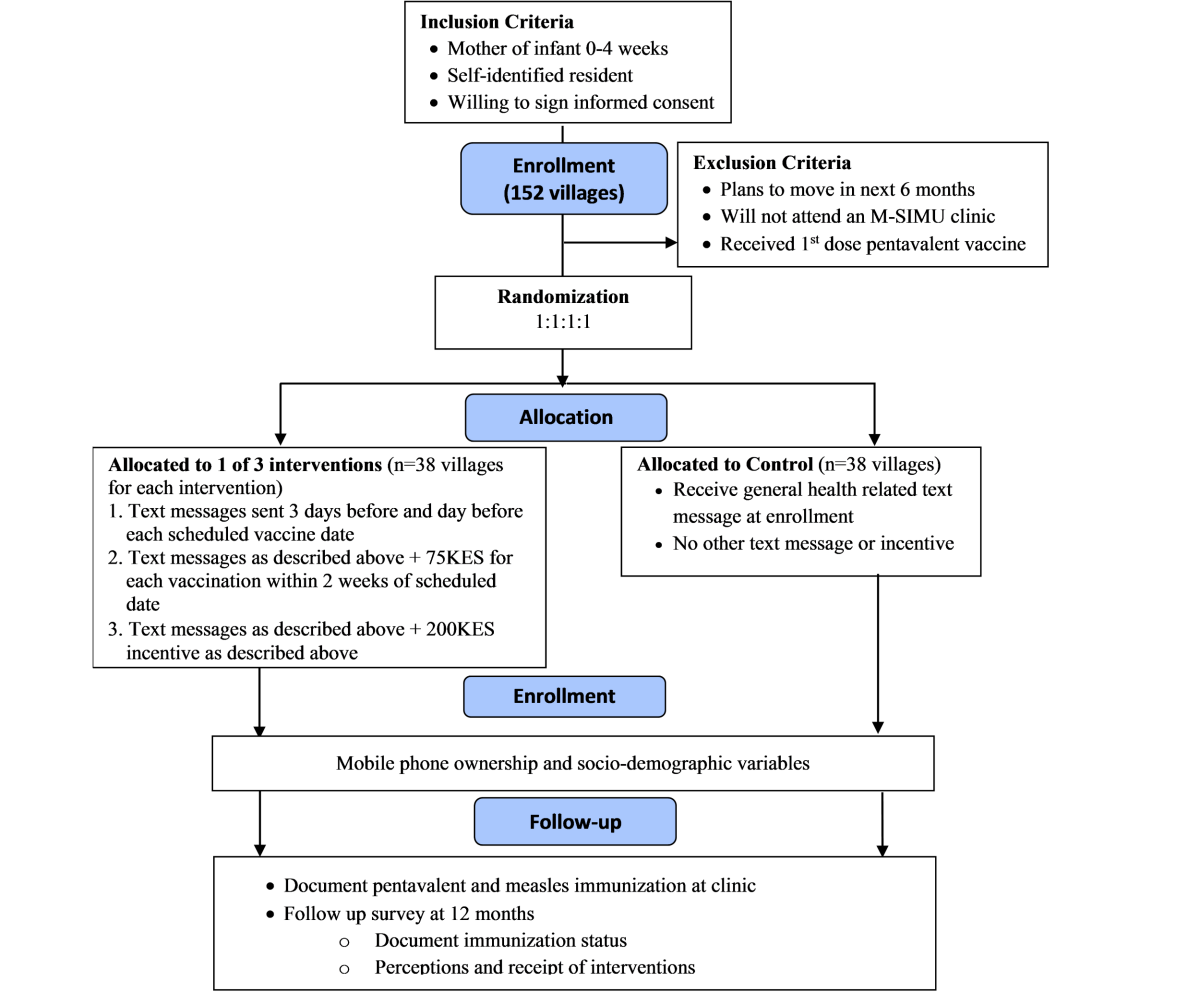
CONSORT diagram of study design.

The M-SIMU study is a four-arm, cluster, randomized controlled trial to evaluate the impact of text message reminders and monetary incentives on pediatric immunization coverage and timeliness in rural western Kenya. Villages, as defined by the Kenyan Medical Research Institute and (KEMRI) and Centers for Disease Control and Prevention (CDC) Health and Demographic Surveillance System (HDSS), are the units of randomization. Villages will be randomized to one of four study arms in a 1:1:1:1 allocation ratio (Figure 1). The study arms include: (1) control, (2) text message reminders, (3) text message reminders plus a 75 Kenyan Shillings incentive (KES; KES85 = US$1 as of August 2015) and, (4) text message reminders plus a KES200 incentive. Text message reminders are sent three days and one day before pentavalent vaccination visits scheduled at 6, 10, and 14 weeks, and measles vaccination at 9 months of age. Incentives are delivered to the participant’s mobile phone if the participant’s child is brought for immunization within 2 weeks of the scheduled date. All eligible mothers/caretakers residing within a study village will be assigned to the study arm that the village was allocated.

### Setting and Participants

The M-SIMU study is located in Rarieda and Gem Districts of Siaya County, Kenya. Malaria, tuberculosis, and human immunodeficiency virus transmission are highly prevalent in this rural setting [33]. In 2008, the under-5 mortality rate was 212 deaths per 1000 live births, with pneumonia and diarrhea as common causes of childhood mortality [13].

The study site is nested within boundaries defined by KEMRI/CDC HDSS. Since 2001, the HDSS systematically collects information on births, deaths, migration, morbidity, and demographics every 4 months for a population of over 220,000 people. Within the HDSS, several disease specific studies have been conducted, including randomized controlled trials for bed-net efficacy, and rotavirus vaccine efficacy [34,35].

Prior to enrollment of the randomized controlled trial, a baseline survey was conducted in the study villages to obtain recent estimates of vaccination coverage for sample size calculation and to collect sociodemographic variables, such as mobile phone ownership and distance to nearest health facility, to perform a restricted randomization of study villages [16]. Additionally, three focus group discussions with 10 to 15 mothers who have a child 12- to 23-months-old were conducted to solicit local and contextual-specific feedback on the content and timing of the text message reminders, incentive amounts, willingness to receive reminders and incentives, and to anticipate any problems or complications associated with delivering text message reminders and incentives.

Villages are included in the cluster randomized controlled trial if they are located within either Gem or Asembo HDSS boundaries. Villages are excluded from the M-SIMU study if there are active immunization intervention/programs (e.g., nongovernmental organizations conducting immunization-related activities, outreach immunization clinics, etc.) that might confound study outcomes. Sample size calculations were conducted to determine the number of villages needed to be able to detect a 15% absolute difference in full immunization coverage at 12 months of age between control and intervention arms.

To ensure accurate population numbers, KEMRI/CDC HDSS casually employs ‘village reporters’ to identify births, deaths, and pregnancies within their community. For the M-SIMU trial, village reporters will be provided a simple mobile phone and trained to send a birth or death notification text message to the RapidSMS server, a free and open-source platform. The notification text message will include the study village and compound number. The RapidSMS server then automatically relays the notification to a field-based Community Interviewer (CI). Following receipt of a birth notification, the CI will visit the newborn’s compound to explain the trial and screen the mother/caretaker for the following eligibility criteria described in Textbox 1.

**Criteria for participant enrollment into the Mobile Solutions for Immunization (M-SIMU) trial.**

Inclusion criteria:

- Mother of infant aged 0- to 4-weeks during the study period
- Current resident of one of the study villages
- Willing to sign informed consent for the study

**Figure 2:**
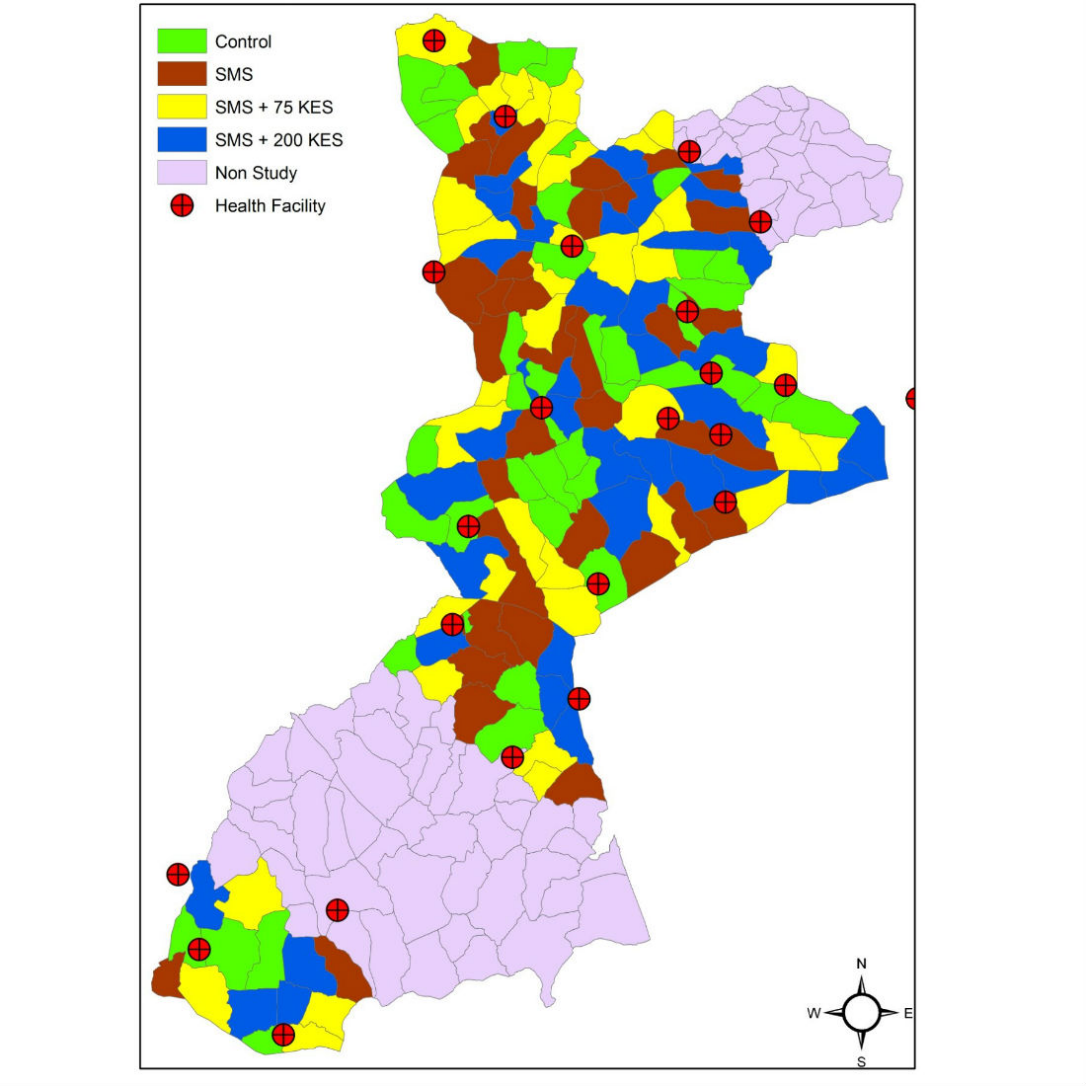
Map of villages coded by study arm.

Exclusion criteria:

- Plans to move out of the study area in the next 6 months
- Has already received immunizations other than birth dose of bacille Calmette-Guerin or polio
- Will not bring infant to an M-SIMU identified clinic (Figure 2)

Mothers are eligible independent of mobile phone ownership. Mothers only need to have access to a mobile phone, whereby access is defined by the mother and could include someone that lives in household, compound, or a neighbor. If no mobile phone can be identified, the mother may use the CI’s phone.

Eligible mothers will be required to provide both oral and written informed consent to the CI. Upon providing consent, the CI will send an enrollment text message to the RapidSMS server containing the mother’s village and compound number, the phone number that can be used to receive text message reminders, the child’s date of birth, the preferred language to receive text message reminders (English, Kiswahili, or Dholuo), and the baby’s first and last name. Upon completion of a successful enrollment text message, the RapidSMS server sends a personalized text message to the mother welcoming her to the study (see Table 1).

### Interventions

The interventions, text message reminders and incentives, are designed to motivate mothers and increase demand for routine pediatric immunizations.

Text message reminders are a component of all three intervention arms and will be sent to the mobile phone number participants identified at enrollment. Text message reminders will be sent using RapidSMS on both 3 and 1 days before the scheduled immunization visits at 6, 10, and 14 weeks for the three doses of pentavalent vaccine and at 9 months for measles as per Kenyan Expanded Programme on Immunization (KEPI) guidelines. Text message reminders will be sent as in English, Kiswahili, or Dholuo language according to the mother’s preference as indicated at enrollment. If a pentavalent vaccination is given later than the scheduled date, then text message reminders for the subsequent pentavalent dose will be reprogrammed to occur at 4 weeks from the date of vaccine receipt, as per KEPI guidelines. As an example, if a child receives pentavalent1 at 8 weeks of age (scheduled to be given at 6 weeks), the immunization reminders for pentavalent2 will be sent when the child is 12-weeks old (instead of the KEPI schedule of 10 weeks).

Text message reminders are composed of a core text and a motivational saying (See Table 1). The core message states which vaccine is due, and if the participant is in an incentive arm, reminds the mother how much money she will receive if the child is vaccinated in a timely manner. The motivational sayings attached at the end of the text message were chosen from the results of focus group discussions held with caregivers of children aged 12- to 23-months old. The four sayings are “Vaccines save Kenyan babies lives,” “Baby < INSERT BABY FIRST NAME> is happy when healthy “Most <INSERT DISTRICT: ASEMBO or GEM> babies get vaccinated, be one of them,” and “Vaccines are available now.” For each vaccine dose, one of the four motivational sayings is randomly selected, with replacement, by the RapidSMS software. The same motivational saying is used for the 3- and 1-days reminder for that particular vaccine dose.

Mobile phone–based monetary incentives are a component of study arms numbers three and four. In both arms, the conditions and delivery of the incentive are identical; only the incentive amount differs. In addition to receiving text message reminders, mothers will receive either KES75 (arm #3;) or KES200 (arm #4) on their mobile phone for each timely dose of pentavalent and measles vaccine, defined as vaccination within 2 weeks of the scheduled date (i.e., pentavalent1 at 6 weeks, pentavalent2 four weeks after pentavalent1 received, pentavalent3 four weeks after pentavalent2 received, and measles at 9 months). If a mother brings her child for vaccination any time 2 weeks after the scheduled date, no incentive will be transferred. Mobile-money incentives will be transferred using the preferred mobile money network of the participant and are aimed to be delivered within 24 hours of a timely vaccination.

**Table 1:** Content of text message reminders sent to participant’s mobile phones.

Message Type	Message Timing	Arm 1: Control	Arm 2 : Reminders Only	Arm 3: Reminders + KES75 Incentive	Arm 4: Reminders + 200KES Incentive
Enrollment message	Enrollment	Thank you for enrolling Baby <BABY’S FIRST NAME> to the KEMRI/CDC M-SIMU study. The greatest wealth is health.	Thank you for enrolling your child in the KEMRI/CDC M-SIMU study. You will get periodic reminders for Baby <BABY’S FIRST NAME>‘s vaccinations. The greatest wealth is health.	Thank you for enrolling your child in the KEMRI/CDC M-SIMU study. You will get periodic reminders for Baby <BABY’S FIRST NAME>’s vaccinations. The greatest wealth is health.	Thank you for enrolling your child in the KEMRI/CDC M-SIMU study. You will get periodic reminders for Baby <BABY’S FIRST NAME>‘s vaccinations. The greatest wealth is health.
3-day reminder message	3 days before EPI due date	No message	Tell Mama <BABY’S FIRST NAME> that <VACCINE NAME> vaccine is due this week. <MOTIVATIONAL MESSAGE>	Tell Mama <BABY’S FIRST NAME>> that <VACCINE NAME> vaccine is due this week. You get KES75 if Baby vaccinated in next 2 weeks. <MOTIVATIONAL MESSAGE>	Tell Mama <BABY’S FIRST NAME> that <VACCINE NAME> vaccine is due this week. You get KES200 if Baby vaccinated in next 2 weeks. <MOTIVATIONAL MESSAGE>
1-day reminder message	1 day before the EPI due date	No message	Tell Mama <BABY’s FIRST NAME> that <VACCINE NAME> vaccine is due this week. Go to the clinic if you haven’t already. <MOTIVATIONAL MESSAGE>	Tell Mama <BABY’S FIRST NAME> that <VACCINE NAME> vaccine is due this week. Go to the clinic if you haven’t already. <MOTIVATIONAL MESSAGE>	Tell Mama <BABY’S FIRST NAME> that<VACCINE NAME> vaccine is due this week. Go to the clinic if you haven’t already. <MOTIVATIONAL MESSAGE>
Motivational message	1 of 4 motivational messages randomly selected and appended to the end of the 3- and 1-day reminders; Motivational message is the same for the 1st and 3rd day reminder of the specific vaccine dose	No message	1. Vaccines save Kenyan babies lives; 2. <Most DISTRICT: ASEMBO OR GEM> babies get vaccinated, be one of them; 3. Baby < BABY’S FIRST NAME > is happy when healthy; or 4. Vaccines are available now.		

The incentive amounts in arms three and four were guided by opinions of mothers, village reporters, and local transport costs. The intent of the incentive is to help offset the costs associated with transportation to the clinic. The transaction costs associated with mobile-money transactions will be borne by the study, such that mothers will receive the full amount indicated. Enrolled mothers will be able to change the mobile phone number for receiving text message reminders and incentives when visiting any M-SIMU clinic.

### Control

Mothers residing in control arm villages will receive a congratulatory text message at enrollment with a general-health related saying, “The greatest wealth is health” (See Table 1). No additional text messages or incentives will be sent to mothers.

At the 12 month follow-up visit conducted at the enrolled mother’s household, a CI will refer mothers of under-vaccinated children from any arm to the nearest clinic.

### Primary Objective

The study is powered to determine if text message reminders, with or without incentives, increase the percentage of fully immunized children (FIC) by 15% as compared with control group children at 12 months of age. A fully immunized child is defined as having received one dose of bacille Calmette-Guerin, three doses of pentavalent and polio vaccines, and one dose of measles vaccine.

### Secondary Objectives

The study assesses several secondary objectives that primarily focus on timely receipt of individual vaccines and effect modifiers on the primary outcome. These secondary objectives include:

1. To determine if FIC coverage measured at 10 months of age varies by study arm;

2. To determine if the proportion of children vaccinated within 2 weeks of each scheduled vaccine date differs by study arm;

3. To determine if the proportion of children who drop-out in vaccination between first and third pentavalent dose differs by study arm;

4. To determine if measles and or pentavalent3 vaccine coverage varies by study arm;

5. To determine if there is a differential effect on vaccine coverage based on mobile phone ownership (owned vs shared) or on residential distance from a health facility.

6. To determine whether other indicators of health status, such as height-for-age, bed-net usage, vitamin A coverage, retention of the maternal and child health card, and all-cause mortality vary by study arm; and

7. To evaluate the direct costs for each intervention arm per additional child vaccinated beyond the status quo (ie, control group).

### Randomization

This constrained randomization was conducted using GAUSS Mathematical and Statistical System. The GAUSS program iterated until 5000 acceptable randomizations were found that met the following criteria: (1) +/− relative 10% over all 152 villages for the means of the variables: full immunization coverage, phone ownership, distance to the nearest clinic, and village population of children 12- to 23- months-old, and (2) +/− relative 25% within each region for the means of the variables: full immunization coverage and phone ownership.

The randomization was also stratified on region such that each study arm contained 30 villages from Gem and eight villages from Asembo region. Data for the randomization came from the baseline survey described previously.

A simple random sample of n=1000 was taken from the 5000 valid randomization sequences. The 1000 sequences were labeled with a three-digit number, 000 to 999. Each sequence allocated 38 villages to one of four groupings (A-D).

Villages were randomized to study arms in a public ceremony attended by location chiefs and Community Advisory Board members on September 12, 2013 (Figure 2). Ten soccer balls were labeled with numbers zero through nine and placed in a cloth sack. Three location chiefs each drew one labeled ball, with replacement, such that a three-digit number, equating to a randomization sequence, was generated. Villages were placed into four groups based on the randomization sequence drawn. Then, four soccer balls labeled with the study arms (#1: control; #2: SMS; #3: KES75; and #4: KES200) were placed in a different cloth sack. A representative from each of the four groupings (A-D) drew one ball, without replacement, to determine the study arm assigned to all villages within the grouping. A similar randomization scheme for a cluster trial was successfully conducted in Zambia [36]. Due to the nature of the intervention and study design, study participants will know their study arm allocation.

### Data Collection

The study is designed to minimally interfere with routine care–seeking behaviors of mothers and routine delivery of care by health practitioners. Participants are interviewed, at most, six times. All participants are interviewed at enrollment when the infant is between 0- and 4-weeks old and at follow-up when the infant is 12-months old. Mothers that bring their child for immunization at an M-SIMU clinic will be interviewed by study staff at each immunization visit (up to four visits).

CIs will administer the enrollment survey using the ODK Collect software on a simple smartphone. Mobile phone literacy, demographics, vaccine perceptions, transportation, and socioeconomic status will be collected at enrollment.

A KEMRI/CDC health facility recorder (HFR) will be stationed at each health facility to document enrolled infants’ immunization. For all enrolled children, the HFR will send a text message to the RapidSMS server. This text message contains the child’s study identification, the date of vaccination, which vaccine was received, and the new phone number if the mother has changed phone lines. If the mother is in an incentive arm (#3 or #4) and the child is vaccinated within 2 weeks of scheduled date, an incentive will be transferred to the phone number designated by the mother. After sending the vaccine receipt text message, HFRs will interview participants to collect means and costs of transportation and other clinic-related expenses for future cost-effective analyses.

For clinics where few immunizations are given per day, there will be no permanent HFR stationed due to financial constraints. Rather, HFRs from neighboring clinics or MOH staff, will visit these smaller clinics at the end of the day, collect immunization information for enrolled mother-infant pairs, and send the vaccine receipt SMS text message to the RapidSMS system. The M-SIMU trial will collect immunization from 24 clinics whose catchment areas envelope study villages (see Figure 2).

When enrolled children reach 12 months of age, CIs will conduct in-home follow up visits to document immunization status using the child’s maternal and child health (MCH) booklet and to collect information on mothers’ perceptions of the intervention(s).

### Data Analysis

The analysis and reporting of results will be conducted in accordance with the **Consolidated Standards of Reporting Trials** guidelines adapted for cluster, randomized trials [37]. A blinded statistician will conduct analyses for primary and secondary outcomes. The primary analyses will be conducted with intention-to-treat principles. The primary outcome, FIC at 12 months of age, will be defined as a binary variable. Risk ratios for achieving FIC by 12 months of age will be calculated for the intervention arms as compared with the control arm. Due to the high number of clusters per study arm, individual level analyses using general estimating equations (GEE) with an exchangeable correlation matrix to account for correlation within clusters will be preferred over cluster level comparisons [38]. As a secondary analysis of the primary outcome, time-to-immunization curves will be constructed using the Kaplan-Meier method and study arms will be compared using the Cox model with frailty or robust variance estimator accounting for cluster. The 25th, 50th, and 75th percentiles for time to immunization and the number of days delayed, in relation to the EPI due date, will also be reported for each vaccine and by study arm. Lastly, adjusted analyses will be conducted if randomization results in imbalanced groups on key demographic variables. Effect estimates will be presented in whole and stratified on mobile phone ownership and clinic proximity. Mobile phone ownership will be defined as a binary variable (‘owns a mobile phone’ or ‘doesn’t own a mobile phone’). Socioeconomic quintile scores will be computed using a multiple correspondence analysis of household assets, livestock, water source, and cooking fuel [39]. Straight-line distances from the participant’s household to the nearest health facility will be calculated using ArcView Geographic Information Systems. An alpha of 0.05 will be assumed for all statistical tests of significance.

### Sample Size

The primary objective is to increase the proportion of infants that are fully immunized at 12 months of age by 15% (eg, a difference between 70% in the control arm and 85% in an intervention arm). There are few interventions available to public health officials that can rapidly raise immunization coverage by this amount so we expect that this size of an effect will represent a meaningful public health impact and could motivate decision makers to adopt this intervention. Type 1 error (alpha) and our power to detect a 15% difference (1-beta) were set to 0.05 and 0.80, respectively.

Village-level immunization coverage estimates were collected during the baseline survey. The baseline survey indicated that approximately 70% (1243/1681) of children were fully immunized by 12 months of age. This estimate was used for assumptions on control arm immunization coverage. Village size (m) was calculated using the most recent HDSS birth cohort data for our study area and assuming 1-year enrollment. Because our sample size per village varies, we computed the harmonic mean for village size, calculated to be 16 children per village. [40].

An important parameter in cluster sample size calculations is *k*, the between cluster coefficient of variation. Although our baseline survey indicated a *k*of 0.14, for sample size calculations we used a conservative estimate of 0.25.

We expect that the percentage of children who will not be enrolled due to not meeting eligibility criteria or refusal will be approximately 10% and the number who will be lost to follow-up after enrollment before 12 months of age, due to death, outmigration, and other reasons, will be 15%. Therefore, we used a 25% reduction from the birth cohort size to determine the number of villages we will enroll in the study.

In order to ascertain a 15% difference between control and intervention arms, 152 villages will be included in the study (38 villages per arm).

### Ethical Considerations

The study protocol received ethical clearance from the Scientific Steering Committee (SSC), the KEMRI-Nairobi Ethical Review Committee (ERC; SSC#2409), Johns Hopkins University Bloomberg School of Public Health (deferred ethical clearance to KEMRI-ERC), and the Centers for Disease Control and Prevention (deferred ethical clearance to KEMRI-ERC). The trial is registered with ClinicalTrials.gov [NCT 01878435, June 10, 2013]. The study’s principal investigators are authors DF and KO from Johns Hopkins University and FO from KEMRI/CDC.

## Results

The baseline survey was conducted March to April 2013 where it was found that 95% (2243/2393) of mothers owned or had access to a mobile phone within the compound. Focus group discussions were conducted in June 2013 to contextualize and adapt the interventions to the community’s needs and to identify the incentive amounts. The randomized-controlled trial completed enrollment in October of 2014. Twelve month follow-up visits to ascertain enrolled participants’ immunization status using the MCH booklet were completed in February, 2016.

## Discussion

Supply side strategies to improve immunization, such as improving cold chain capacity, increasing procurement, and staff training, have been effective, but immunization gaps persist. Demand side interventions, such as text message reminders and incentives, that target cost of transport, vaccine fears and perception, and forgetfulness may play a more prominent role in reaching the “last mile” with timely immunizations.

Despite a lack of rigorous scientific evidence of effectiveness, mHealth and conditional cash transfer programs continue to spread throughout Africa [41-43]. This is the first randomized study to evaluate the impact of text message reminders and mobile money incentives on immunization coverage within sub-Sahara Africa. Evidence generated by this project will assist decision makers in the Kenyan Ministry of Health, as well as those in other African countries, before committing the investment, time, and effort that will be necessary to scale-up these programs. Moreover, this project has the opportunity to demonstrate the potential of mobile phone technologies in achieving the Millennium Development Goal of reducing childhood mortality in Africa. We anticipate results in 2016.

## Appendix

Multimedia Appendix 1 Peer review summary report from scientific steering committee.

## Abbreviations

CDC: Centers for Disease Control and Prevention
CI: community interviewer
ERC: ethical review committee
FIC: fully immunized child
GEE: general estimating equation
HDSS: health and demographic surveillance system
HFR: health facility recorder
KEPI: Kenyan Expanded Programme on Immunization
KES: Kenyan Shilling
M-SIMU: Mobile Solutions for Immunization
MCH: maternal and child health
SSC: scientific steering committee
