# Supplementary material for: The Mobile Solutions for Immunization (M-SIMU) Trial: A Protocol for a Cluster Randomized Controlled Trial That Assesses the Impact of Mobile Phone Delivered Reminders and Travel Subsidies to Improve Childhood Immunization Coverage Rates and Timeliness in Western Kenya
Source: JMIR Res Protoc. 2016 May 17;5(2):e72. doi: 10.2196/resprot.5030 (PMC4887657; doi:10.2196/resprot.5030)
Supplement: Multimedia Appendix 1 [file resprot_v5i2e72_app1.pdf]

KENYA MEDICAL RESEARCH INSTITUTE
CENTRE FOR GLOBAL HEALTH RESEARCH
01 OCT 2012
P.O. Box 1578, KISUMU

KEMRI
KENYA MEDICAL RESEARCH INSTITUTE

KENYA MEDICAL RESEARCH INSTITUTE

P.O. Box 54840-00200, NAIROBI, Kenya
Tel (254) (020) 2722541, 2713349, 0722-205901, 0733-400003; Fax: (254) (020) 2720030
E-mail: director@kemri.org info@kemri.org Website:www.kemri.org

ESACIPAC/SSC/100918 **27th September, 2012**

Danny Feikin

Thro'
Director, CGHR
KISUMU

3/10/2012

REF: SSC No. 2409 (Revised) – Randomized controlled trial of the impact of mobile phone delivered reminders and travel subsidies to improve childhood immunization coverage rates and timeliness in Western Kenya.

This is to inform you that the above-mentioned protocol in which you are the PI was reviewed by KEMRI SSC and it was recommended that you revise this proposal in view of the attached suggestions made by our reviewers.

Kindly submit the revised protocol within one (1) month from the date of this letter i.e., 28th October, 2012.

You are advised to submit **5 copies** of the revised proposal.

Sammy Njenga, PhD
SECRETARY, SSC

Encl(s)

Additional comments:

Please provide a comprehensive list of typed comments you may wish to be sent anonymously to the PI of the protocol

---

1. On page 7 Investigators advance several reasons why they believe their approach will be successful, suggesting they been with a mindset that the study will be a success!!!
2. Investigators rightly note that if findings show that either small subsidies and or SMS reminders improve coverage, the experience might be applicable to other African Countries exploring strategies to improve coverage.

   Therefore, the proposed project has huge unrecognized IP potential, if among study outputs, a book/booklet can be written by the Investigators to document this novel experience. Such a booklet is likely to be highly sought in the global market. This has potential to provide copyrights for the investigators, CGHR, PHHSRP and KEMRI as per KEMRI IP policy.
